# Parent-dependent stressors and the onset of anxiety disorders in children: links with parental psychopathology

**DOI:** 10.1007/s00787-017-1038-3

**Published:** 2017-08-08

**Authors:** Jennifer L. Allen, Seija Sandberg, Celine Y. Chhoa, Tom Fearn, Ronald M. Rapee

**Affiliations:** 1 Department of Psychology and Human Development, UCL Institute of Education, University College London, 20 Bedford Way, London, WC1H 0AL UK; 2 Mental Health Sciences Unit, University College London, London, UK; 3 Department of Statistical Science, University College London, London, UK; 4 Centre for Emotional Health, Macquarie University, Sydney, NSW Australia

**Keywords:** Child anxiety, Stress, Life events, Chronic adversity, Parent psychopathology, Stress generation

## Abstract

Exposure to stressors is associated with an increased risk for child anxiety. Investigating the family origins of stressors may provide promising avenues for identifying and intervening with children at risk for the onset of anxiety disorders and their families. The aim of this study was to compare the frequency of parent-dependent negative life events and chronic adversities experienced by children with an anxiety disorder (*n* = 34) in the 12 months prior to the onset of the child’s most recent episode, compared to healthy controls (*n* = 34). Life events and chronic adversities were assessed using maternal report during an investigator-based interview, which provided independent panel ratings of the extent that reported experiences were related to parent behaviour. There were no group differences in the number of parent-dependent negative life events for anxious children compared to controls. However, significantly more parent-dependent chronic adversities were present for anxious children compared to controls. Findings suggest that parents contribute to an increased frequency of chronic adversities but not negative life events prior to their child’s most recent onset of anxiety. Furthermore, increased child exposure to parent-dependent chronic adversities was related to parental history of mental disorder.

## Introduction

Stressful life events have been identified as an important risk factor for childhood anxiety disorders [1, 2]. For some children, the link between stressor exposure and anxiety may be direct, such as the emergence of specific phobia of dogs following an attack, or experiences of bullying and social rejection leading to social anxiety disorder, consistent with classical conditioning accounts of anxiety development (e.g., Rachman [3]). However, contemporary theory also predicts that exposure to any form of stressors may contribute to the development and maintenance of anxiety due to increased child perceptions of threat and uncontrollability over adverse circumstances [4]. Exposure to stressors may result in environmental changes known to promote child anxiety, such as parental overprotection due to increased perceptions of child vulnerability [1]. Features associated with an anxious, inhibited temperament including emotional reactivity, threat processing biases, and an avoidant coping style may also increase child vulnerability to the impact of adversity. Early life stress is related to changes in the child’s developing stress response systems [e.g., dysregulation of the hypothalamic–pituitary–adrenal (HPA) axis], resulting in sensitization to future stressors such that lower levels of stress are required to provoke the onset of anxiety in children who have experienced previous adversity [5]. Thus, from a theoretical perspective, a better understanding of the origins of stressors experienced by anxious children is important for the prevention of a continuous cycle of stressors and symptoms.

In the life events literature [6], a distinction is made between stressors that are time limited with an identifiable onset and ending (negative life events) as well as those that are more enduring in nature (chronic adversities). Life events (e.g., death of a loved one, natural disasters, failing an important exam) are, therefore, discriminated from chronic adversities (e.g., chronic illness, bullying, family conflict) in that the former experiences are time limited, even though their consequences may be ongoing. This distinction may be important given claims that the two types of stressors may play a different role in the course of different disorders, with discrete life events triggering the onset of episodic disorders such as depression, and chronic adversities showing a stronger relationship with childhood-onset anxiety and chronic anxiety disorders [7]. There is substantial evidence for increased rates of negative life events and chronic adversities for anxious children compared to healthy controls [8–10]. Importantly, these anxious–control differences remain when controlling for the presence of externalizing and depressive disorders [11, 12]. Most research investigating anxious/control differences has utilised a time frame that identifies stressors occurring 12 months prior to the date the study was conducted. This method does not take into account when the actual disorder had its onset and therefore the relationship between stressor occurrence and onset of disorder varies widely between participants. A more consistent method is to specify the occurrence of stressors within a period of 12 months prior to onset of the anxiety disorder. Such studies have also shown increased rates of both negative life events and chronic adversities for anxious children relative to controls [13–15]. These studies provide stronger evidence for a causal role of stressors in triggering the onset of clinical episodes.

Despite the well-established link between increased rates of stressors and anxiety in children, there is little research on the origins of these stressors. This is an important focus of study, because if all stressors are truly random (e.g., natural disasters), then there is little that can be achieved by changes in policy or other forms of intervention [16]. However, if stressors are related to factors that are amenable to change, this may provide an important avenue for prevention and treatment. Transactional models of stress and psychopathology recognize the potential role of parents in ‘stress generation’, acknowledging that stressors that exert a negative impact on children may be related to parent behaviour (e.g., marital conflict, unemployment) [17, 18]. These models differentiate between stressors that are independent of an individual’s behaviour (e.g., death or illness of a family member) and those that are wholly or partly behaviour related (e.g., interpersonal conflict). Parent behaviour-dependent stressors are believed to elicit anxiety due to parental modelling of anxiety, withdrawal and avoidant coping, child concern for family difficulties, or their impact on the parent–child relationship and/or parenting practices [19]. Parents who suffer from a mental disorder are more likely to behave in ways that contribute to the occurrence of stressors, therefore, theory has identified parent behaviour-dependent stressors as a potential mediator of the relationship between parental psychopathology and anxiety in children [2]. Theories of parenting stress [20, 21] highlight the reciprocal nature of this relationship: parenting stress is associated with parental psychopathology and parents with mental health issues tend to experience more intense reactions to stressful events. In turn, parenting stress and psychopathology both influence parental behaviour and feelings towards their child (e.g., overprotection, negativity), serving to further promote anxious vulnerability.

Few studies have examined the association between parental psychopathology and stress generation for anxious children. This is surprising given the increased rates of mental disorders including anxiety, depression, and substance abuse in the parents of anxious children [22, 23], and evidence that parental psychopathology places children at increased risk for parent behaviour-related stressors, such as interparental conflict [24]. Only one study has previously reported on relations between parental psychopathology and stressful life events prior to the onset of anxiety disorders in children. Goodyer et al. [14] interviewed mothers of children aged 8–16 years (*n* = 100) concerning stressful life events 12 months prior to the recent onset of anxiety, and mothers of matched controls (*n* = 100) about life events during an equivalent time period. Findings indicated that maternal distress, maternal adversity and stressful life events exerted independent, but additive effects on the likelihood of being in the anxious versus control group. However, assessment of maternal adversity was limited to poor confiding relationships, and only maternal, but not paternal psychopathology was examined. Fathers are viewed as playing an important and differing role in the aetiology of anxiety to mothers [25, 26]. Fathers teach children to be confident, brave and independent, while the maternal role has a stronger emphasis on protection and care [25, 26]. The presence of psychopathology is likely to impair the ability of mothers and fathers to perform these roles in such a manner as to promote child autonomy and the ability to regulate negative emotions such as anxiety [2, 27]. Investigating the relationships between both paternal and maternal history of mental disorder, parent behaviour-related stressors and the onset of anxiety in children is important given that research on the development of anxiety has neglected the role of fathers, and may also help inform the nature and focus of maternal versus paternal involvement in family intervention [28].

The present study aims to examine the number of parent-dependent life events and chronic adversities experienced by anxious children prior to the onset of their most recent clinical episode in comparison to matched controls over an equivalent time period. To date, research has focused solely on life events that are related to child behaviour [11, 12, 28]. The current sample overlaps with a previous case–control comparison of stressors prior to the onset of anxiety [13]. However, this previous study did not examine relations between parental psychopathology, parent-dependent stressors and anxiety in children. We used an investigator-based interview to gather extensive information about reported experiences from mothers, thereby allowing (1) a panel of experts to determine whether reported experiences met criteria for a life event or chronic adversity, (2) comparison of maternal (subjective) and independent panel (contextual) impact ratings, and (3) independent ratings of the extent of dependence of stressors on parent behaviour. It was predicted that compared to matched controls, anxious children would experience significantly more (1) parent-dependent negative life events, and (2) parent-dependent chronic adversities. It was hypothesized that child anxiety symptoms and maternal distress would be significantly related to increased rates of parent-dependent life events and chronic adversities. Finally, we predicted that parental history of psychological disorders would be associated with significantly more parent-dependent negative life events and chronic adversities.

## Methods

### Participants

A total of 68 children aged 7–12 years and their mothers participated in this study (see Table 1). All mothers were the child’s biological mother and primary caregiver. The anxious sample comprised 34 children and their mothers who formed consecutive admissions to the Centre for Emotional Health Clinic, Macquarie University, Sydney, Australia. All children met DSM-IV criteria [29] for a primary diagnosis of an anxiety disorder. Children were excluded if a non-anxiety diagnosis was present prior to their initial onset of an anxiety disorder. The control sample comprised 34 children and their mothers, matched to an anxious child on sex and age. Children were included in the control group only if they did not meet DSM-IV criteria for any mental disorders [29]. The control sample was recruited via advertisements in school bulletins asking for happy, confident children and their mothers to serve as a comparison group, and received AUD$50 for their participation. Five parent–child dyads from single-parent families and their matched pair from the other group were excluded from the original sample [13] to ensure that findings could not be attributed to differing family structures.

**Table 1:** Group differences in demographic characteristics and parent mental disorder

	Anxious	Control	*t*/*χ* ^2^
Maternal age			
*M* (SD)	39.74 (5.13)	41.62 (1.84)	−1.56
Number of siblings			
*M* (SD)	1.50 (0.96)	1.71 (0.84)	−0.942
Australian % (*n*)	82 (28)	71 (24)	1.308
Mother completed 12 years of education % (*n*)	88 (30)	88 (30)	–
At least one parent employed full-time % (*n*)	91 (31)	100 (34)	3.138
Household income % (*n*)			
<$40,000	9 (3)	3 (1)	3.730
$40,000–$80,000	52 (17)	35 (12)	
>$80,000	39 (13)	62 (21)	
Maternal history of mental disorder % (*n*)	65 (22)	12 (4)	20.18***
Paternal history of mental disorder % (*n*)	27 (9)	9 (3)	3.643

Some percentages may add up to more than 100% due to rounding up

* *p* < 0.05, ** *p* < 0.01, *** *p* < 0.001

## Measures

### Family background questionnaire

A brief family socio-demographic questionnaire assessed child age, gender, nationality, siblings, maternal age, education, parental employment, family income and if parents had seen a mental health professional.

### Spence children’s anxiety scale: child and parent versions (SCAS-C/P)

Child and parent versions of the SCAS [30, 31] were used to assess child anxiety symptoms. The SCAS-C/P consists of six scales that reflect symptoms of anxiety disorders included in the DSM-IV [29]. The SCAS-C/P asks respondents to rate how often each of 38 statements applies on a four-point scale from 0 (never) to 3 (always). The SCAS-C/P total scores are calculated by summing all subscale scores. Both child and parent versions of the SCAS have been shown to be reliable and valid measures of child anxiety symptoms [30, 32]. Cronbach’s alphas were 0.94 for SCAS-P scores and 0.86 for SCAS-C scores in the current sample.

### Depression anxiety stress scales—brief version (DASS-21)

The DASS [33] assessed maternal report of emotional distress during the past week. The 21-item DASS is a brief version of the original 42-item DASS [33]. It consists of three 7-item scales assessing anxiety, depression and stress which are summed to form a total DASS-21 score. Each item is rated on a four point scale (0 = ’Did not apply to me at all’ to 3 = ’Applied to me very much, or most of the time’). The DASS has excellent internal consistency, validity and reliability [33, 34]. Alpha for DASS total score was 0.92.

### Anxiety disorders interview schedule for DSM-IV: child and parent versions (ADIS-IV-C/P)

The ADIS-IV-C/P [35] are semi-structured clinician-administered interviews that assess anxiety and other common mental disorders in children aged 6–17 years according to DSM-IV criteria [29]. The ADIS-IV-C/P provide clinician-based consensus child diagnoses for primary (most interfering) and secondary diagnoses (all other disorders where DSM-IV criteria are met) on the basis of child and parent report of symptoms and associated impairment. The ADIS-C/P has good psychometric properties [36]. Our clinic has found good to excellent inter-rater agreement for child anxiety diagnoses, with kappas ranging from 0.68 to 0.93 [37].

### Psychosocial assessment of childhood experiences: parent version (PACE)

The frequency, type, timing, impact and independence of life events and long-term psychosocial events experienced by children was assessed using maternal report on the PACE [38, 39], a standardized investigator-based interview. The PACE is based on the life events and difficulties schedule (LEDS) [40], an investigator-based interview of adult life events. The PACE follows the format and principles of the LEDS, with modifications to event coverage and re-conceptualization of event impact ratings for children. Experiences covered by PACE include: household moves, changes in household membership, child separations from family members, health and illness, accidents and hospital visits, family and peer relationships, marital events, witnessing or experiencing a traumatic event, significant achievements and exceptionally good experiences.

PACE elicits information about the circumstances surrounding each reported experience (e.g., family and social context), as well as the occurrence and timing of each experience. This information is used to decide whether each reported experience meets criteria for a life event, on the basis that most children of the same age as the participant would find the experience either (1) threatening, unpleasant or upsetting or (2) very pleasant, enhancing to self-esteem or opening up new opportunities. Contextual threat of life events is assessed by a panel of trained raters using a four-point scale (0–3), with higher scores indicating greater threat. Parent (subjective) ratings of life event impact are similarly assessed. Examples of life events rated as a low threat (1) by panel experts included a parent’s trip overseas and changes in a parent’s working hours. Life events rated as moderate high threat (2 or 3) included a parent starting full-time work and learning of a parent’s previous marriage. PACE also assesses a variety of long-term experiences (LTEs), conceptualised as the long-term equivalent of life events. PACE covers LTEs likely to have a negative impact (e.g., health problems, parent conflict) or a positive impact (e.g., a hobby or position of responsibility). Chronic adversities are defined as LTEs that lasted for a minimum of 4 weeks and exerted an objective negative impact on the child (panel-rated score of at least 1 on a four-point scale from 0–3). Examples of chronic adversities rated as low threat (1) by panel experts included a family’s long-term stay with a relative, and parent conflict over the child’s behaviour. Chronic adversities rated as moderate or high threat (2 or 3) included parent conflict with the child’s teacher and parental conflict. PACE has shown good validity for the report of life events and LTEs during 12- and 18-month time frames [39, 41]. The current study applied a 12-month time frame, consistent with previous research examining links between stressors and the onset of anxiety in children [13, 14].

PACE assesses the independence of each life event and LTE in relation to child and parent behaviour rather than in relation to symptoms of psychopathology, as in the LEDS [40]. This change was deemed necessary because of the possibility that life events may stem from an individuals’ personal qualities (e.g., personality, motivation) as well as from the mental disorder per se [42]. The independence of life events and LTEs are rated by the panel on a four-point ordinal scale, from 1 “totally independent” to 4 “totally behaviour related” to parents’ behaviour and the child’s behaviour. A life event was only considered parent-dependent if it was rated i) “totally” or “probably” related to the behaviour of parents and ii) “totally” or “probably” independent of the behaviour of the target child. This was to ensure that only life events and LTEs unrelated to child behaviour and/or anxiety symptoms were included in the analyses. Frequency counts for parent-dependent negative life events and LTEs were obtained by combining the “totally” and “probably behaviour-related” categories, therefore, only stressors that are likely to have been caused by the parent’s behaviour were included in the analyses. Positive life events and LTEs were excluded from analysis as no existing theoretical models have identified these factors as contributors to the aetiology of anxiety in children.

### Parent history of mental disorder

PACE includes an assessment of the lifetime history of mental disorder for the target child’s parents. If the respondent affirms that she or the child’s father has ever experienced depression, anxiety or any other mental disorder, then the PACE interviewer administers further questions concerning the timing of the disorder, whether treatment was sought/received, and the nature of treatment (e.g., medication, counselling services, general practitioner, mental health clinic, hospital as an in-patient or outpatient). The nature and extent of impairment due to any reported mental disorder is assessed in a range of areas including the parents’ daily routine, household chores, family and peer relationships, work and social life. The absence of significant symptoms and no treatment or related social impairment is coded 0 (none); symptoms but no accompanying treatment or related social impairment is coded 1 (possible); and treatment or social impairment related to symptoms of mental disorder is coded 2 (definite). The categories of ‘possible’ and ‘definite’ were combined because the number of ‘possibles’ (mothers: *N* = 4; fathers: *N* = 5) were too small to allow separate estimation of the effects of this category (see Table 1). PACE assessment of parental history of mental disorder indicated moderate to substantial agreement with parent questionnaire report of having ever seen a mental health professional (mothers: kappa = 0.64, fathers: kappa = 0.49). Maternal history of mental disorder was also significantly related to maternal self-reported current (past week) emotional distress (*r* = 0.49).

## Procedure

Following the receipt of institutional ethics approval, mothers were sent information and consent forms. Mothers who provided written informed consent were mailed questionnaires prior to the interview to return at the time of the clinical assessment. Mothers of anxious children were interviewed by the first author to ascertain the timing of onset. Onset was defined as the occurrence of the child’s most recent clinical episode, requiring the presence of clinically significant anxiety and related impairment. Mothers were only informed that they would be interviewed about stressors preceding onset after the timing of onset was established. Significant dates (e.g., school term times, national holidays) and personal landmarks (e.g., birthdays, changes in residence) were marked on a timeline to assist mothers with dating onset. During the interview, additional events were recorded on the timeline and used as memory cues to further facilitate the accurate dating of reported experiences.

Mothers of anxious children completed PACE interviews to assess stressors in the 12-month period prior to the onset of their child’s most recent clinical episode. The average period from the onset of the most recent anxiety episode to the date of the PACE interview was 4.46 months (SD = 3.83, range 1–14 months). Therefore, the time period over which mothers were asked to recall events ranged from 13 to 26 months (median 15 months). Mothers of controls reported on an equivalent time period to the mother of their matched anxious counterpart. That is, a mother of a control child matched to an anxious child whose onset was 3 months prior to their mother’s PACE interview would be asked to report on experiences dating from 3 to 15 months prior to the time of their own PACE interview. One-tailed Spearman’s correlations between the length of time from the PACE interview to the date of onset and the number of parent-dependent negative life events (calculated on the basis of separate maternal and panel ratings) and chronic adversities were all non-significant (all *p*s < 0.05), indicating that there was not a significant drop-off in recall. Mothers were also the sole informant for their own history and paternal history of mental disorder during the PACE interview.

Interviews were conducted by the first author, who completed extensive training with one of the developers of the PACE interview. Training included practice in rating interviews using volunteers and videotaped interviews, guided by manual, dictionary of life event examples and PACE Training Materials [43, 44]. The panel meetings consisted of two panellists: the PACE interviewer (first author) and a second independent rater with substantial experience in the panelling process [39]. During these meetings, the panellists came to consensus agreement as to whether each experience qualified as an event in addition to rating each reported experience on contextual threat and independence. In the few instances where panellists disagreed (<5), the rating of the second panellist was recorded due to her more extensive experience with the PACE panelling process. The second panellist was blind to subjective information, such as child and parent reactions to event(s) and child diagnostic status. Following standard PACE procedure, decisions regarding whether each reported experience met criteria to be classified as a chronic adversity were made during the panelling process, precluding the assessment of maternal impact ratings for chronic adversities.

### Inter-rater reliability for panel ratings

Panel ratings were compared to those provided by a trained external rater for a randomly selected subset of 25% of the sample to determine inter-rater reliability. This rater was presented with the written synopses excluding subjective information (e.g., child’s reaction to the reported experience) and was blind to child diagnostic status and parental history of mental disorder. Absolute agreement between panel ratings and those of the external rater were calculated using the intraclass correlation coefficient (ICC). ICCs were .81 for parent-dependent negative life events and .90 for parent-dependent chronic adversities.

### Statistical analyses

We first present descriptive statistics and between-group comparisons on demographic data, child anxiety and parental history of mental disorder. Groups were compared using Chi squares and two-tailed independent samples t tests. We then investigated associations between life event variables, child anxiety and parental psychopathology using Spearman’s rank correlation coefficients (*rho*) given that visual inspection of histogram plots and data summaries indicated that data for some life events and chronic adversities variables were skewed. Next, we conducted separate Poisson regression models with robust standard errors to determine group differences in the relative frequency of parent-dependent life events and chronic adversities. Finally, we used separate Poisson regression models with robust standard errors to investigate the association between parental history of mental disorder and the frequency of parent-dependent chronic adversities in the whole sample. Poisson regression is the standard approach to investigate the relationship between a dependent variable that is a count of events that has, in principle, an unlimited range and one or more explanatory variables. No covariates were included in the regression analyses. All analyses were conducted using SPSS 22 [45].

## Results

### Demographic data, child and parental psychopathology in the anxious and control groups

The anxious sample comprised 34 children (50% males; *M* = 9.47 years, SD = 1.71) and their mothers. Primary diagnoses for children were as follows: generalized anxiety disorder 53%, separation anxiety disorder 26%, social phobia 9%, specific phobia 6% and obsessive–compulsive disorder 6%. For the majority of children their current difficulties represented a recurrent episode, with only six children experiencing the first onset of an anxiety disorder. Most children (85%) met criteria for more than one anxiety disorder and 21% met criteria for a non-anxiety diagnosis, including dysthymia (*n* = 3) and externalising disorders (*n* = 4). Twenty-two mothers reported a lifetime history of mental disorder, including depressive disorders (*n* = 13), anxiety disorders (*n* = 8) and alcohol use/dependence (*n* = 1) on the basis of the PACE interview [38, 39]. Nine fathers were reported to have a lifetime history of depression (*n* = 6), substance use/dependence (*n* = 2) or an anxiety disorder (*n* = 1).

The control sample comprised 34 children (50% males) aged between 7 and 12 years (*M* = 9.47 years, SD = 1.54) and their mothers. Control children were matched to anxious children on sex and age within 4 months of the birth date of the anxious child (*M* = 1.94 months, *SD* = 1.43). Control children were screened for the presence of mental disorders using the ADIS-IV-C/P [35]. Four mothers of controls reported a lifetime history of a depressive disorder (*n* = 2), anxiety disorder (*n* = 1) and psychosis (*n* = 1), while three fathers had a lifetime history of depression (*n* = 1), anxiety (*n* = 1) or alcohol use/dependence (*n* = 1), assessed using PACE [38, 39]. The anxious and control samples did not differ significantly on any socio-demographic variables, but mothers of anxious children were significantly more likely to report a history of mental disorder than mothers of controls (see Table 1).

### Group differences in child anxiety symptoms and parental psychopathology

Table 2 presents the means and standard deviations for both samples on clinical measures. Significantly more mothers of anxious children reported a possible or definite history of a mental disorder (*n* = 22) than mothers of controls (*n* = 4). In contrast, there was no significant difference in the number of fathers of anxious children (*n* = 9) and controls (*n* = 3) regarding paternal history of mental disorder (see Table 1). Anxious children displayed more severe anxiety than controls on the basis of child self-report and maternal report of anxiety symptoms. Mothers of anxious children reported significantly greater emotional distress than mothers of controls.

**Table 2:** Group differences in maternal emotional distress and child anxiety symptoms

	Anxious	Control	*t*
SCAS-P			
*M* (SD)	36.97 (15.05)	8.15 (6.08)	10.353**
SCAS-C			
*M* (SD)	37.76 (15.47)	12.82 (8.44)	8.252**
DASS			
*M* (SD)	30.55 (18.69)	12.12 (7.86)	5.288**

*SCAS-P* Spence children’s anxiety scale: parent version [31], *SCAS-C* Spence children’s anxiety scale: child version [30], *DASS* depression and anxiety stress scales (Lovibond and Lovibond 1995)

** *p* < 0.01, two-tailed

### Correlations between child anxiety symptoms, maternal emotional distress and parent-dependent negative life events and chronic adversities

Maternal report of parent-dependent negative life events were significantly related to panel-assessed parent-dependent negative life events, *r* = .84, *p* < .001. Parent-dependent chronic adversities were significantly related to more severe child anxiety symptoms (SCAS-C/P) and greater maternal emotional distress. However, no significant associations were present between child anxiety symptoms or maternal emotional distress and parent-dependent negative life events. Table 3 presents one-tailed Spearman’s correlations between maternal and child self-report of anxiety symptoms, maternal emotional distress and life event variables.

**Table 3:** Spearman’s correlations between child anxiety symptoms, maternal emotional distress and parent-dependent negative life events and chronic adversities

	SCAS-P	SCAS-C	DASS
Parent-dependent negative life events			
Maternal ratings	0.12	0.08	0.14
Panel ratings	0.05	0.07	0.18
Parent-dependent chronic adversities	0.39***	0.39***	0.35**

*SCAS-P* Spence children’s anxiety scale: parent version [31], *SCAS-C* Spence children’s anxiety scale: child version [30], *DASS* depression and anxiety stress scales (Lovibond and Lovibond 1995)

** *p* < 0.01, *** *p* < 0.001, one-tailed

### Anxious vs. control group membership as a predictor of the frequency of parent-dependent negative life events and chronic adversities

Table 4 presents the estimates of rate ratios for the anxious and control groups. PACE interview identified a total of 227 life events and 160 chronic adversities. Of these, 28 life events (maternal ratings), 33 life events (panel ratings) and 51 chronic adversities (panel rated) were classified as dependent on the parent’s behaviour. Poisson regression analyses indicated that there was no significant difference between anxious and control children in the number of parent-dependent negative life events. In contrast, there was a significant effect of group on the number of parent-dependent chronic adversities, with an extra 136 parent-dependent chronic adversities for anxious children for every 100 such events in the healthy control group (RR = 2.36, 95% CI 1.62–4.40; *p* < 0.01).

**Table 4:** Anxious vs. control group membership as a predictor of the frequency of parent-dependent negative life events and chronic adversities from regression models

	Anxious *M* (SD)	Control *M* (SD)	*B*	SE	RR	95% CI
Parent-dependent negative life events						
Maternal ratings	0.53 (0.83)	0.29 (0.58)	0.59	0.39	1.80	0.83–3.90
Panel ratings	0.50 (0.83)	0.47 (0.79)	0.06	0.35	1.06	0.54–2.10
Parent-dependent chronic adversities	0.97 (1.38)	0.41 (0.86)	0.86	0.32	2.36	1.62–4.40**

** *p* < 0.01, *df* = 66

### Parental history of mental disorder and the frequency of parent-dependent negative life events and chronic adversities

Table 5 presents the adjusted estimates of rate ratios for maternal and paternal history of mental disorder. Regression analyses indicated a significant effect of maternal history of mental disorder on parent-dependent negative life events (maternal ratings), with an extra 191 parent-dependent negative life events in the history group for every 100 total life events in the no-history group (RR = 2.91, 95% CI 1.35–6.27, *p* < 0.01). Similarly, there was a significant effect of maternal history of mental disorder on parent-dependent negative life events (panel ratings), with an increase of 149 parent-dependent negative life events in the history group for every 100 events in the no-history group (RR = 2.49, 95% CI 1.19–5.20, *p* < 0.05). In contrast, regression analyses indicated no significant effect of paternal history of mental disorder on parent-dependent negative life events, based on maternal or panel ratings (*p*s > 0.05). However, there was a significant effect of both paternal and maternal history of mental disorder on the frequency of parent-dependent chronic adversities, with increase of 245 (246) chronic adversities assessed for children whose mother (father) had a lifetime history of mental disorder for every 100 total chronic adversities in the no-history group (RR = 3.45, 95% CI 1.66–7.17, *p* < 0.001; RR = 3.46, 95% CI 1.73–6.93, *p* < 0.001, respectively).

**Table 5:** Parental history of mental disorder as predictors of parent-dependent negative life events and chronic adversities

	Maternal History of Mental Disorder				Paternal History of Mental Disorder			
	*B*	SE	RR	95% CI	*B*	SE	RR	95% CI
Parent-dependent negative life events								
Maternal Ratings	1.07	0.39	2.91	1.35–6.27**	−0.58	0.64	0.56	0.16–1.95
Panel Ratings	0.91	0.38	2.49	1.19–5.20*	−0.76	0.66	0.47	0.13–1.70
Parent-dependent chronic adversities	1.24	0.37	3.45	1.66–7.17***	1.24	0.35	3.46	1.73–6.93***

* *p* < 0.05,** *p* < 0.01,*** *p* < 0.001, *df* = 66

## Discussion

Contrary to our hypothesis, anxious children did not experience significantly more parent-dependent negative life events in the 12 months preceding the onset of their most recent episode compared to matched controls. This finding was consistent regardless of whether life events were assessed based on maternal or contextual ratings of life event impact. However, findings did support the prediction that significantly more parent-dependent chronic adversities would be present for anxious children prior to onset compared to controls. Results examining linear relationships between main study variables mirrored the group findings, with child anxiety symptoms significantly related to parent-dependent chronic adversities, but not life events. Our results are consistent with those of Goodyer et al. [14], who found that maternal adversity exerted an independent effect on anxious (versus control) group membership. It is unclear why anxious children appear to encounter more parent-dependent chronic adversities, but not life events prior to the onset of their difficulties. Phillips et al. [7] suggested that life events may be more relevant for triggering the onset of episodic disorders such as depression, whereas chronic stressors predict early-onset anxiety disorders and chronic anxiety. The stronger links between chronic adversities and child anxiety as opposed to life events suggest that increased rates of parent-dependent chronic adversities may stem from the relatively stable characteristics of parents or their social environment [13]. For example, parent behaviour-related adversities may reflect the quality of parents’ relationships (e.g., conflict with the child’s siblings or extended family members), their broader environment (e.g., stressful work environment, disadvantaged neighbourhood), or parental psychopathology—a possibility explored in more detail below.

In partial support of our hypothesis, significant associations were present between parent-dependent negative life events with maternal, but not paternal history of mental disorder. The cross-sectional design of this study does not allow us to draw any conclusions concerning the direction of this association. Mothers may be more likely to behave in ways that increase stressor occurrence, perhaps due to the greater parenting role strain reported by mothers compared to fathers [46]. Alternatively, mothers may be at increased risk for psychopathology when their child is exposed to parent-dependent life events, given that women report a greater emotional impact of interpersonal stressors [47]. Interestingly, findings of a family history study suggest that elevated risk for anxiety disorders in the children of anxious parents appears to be largely confined to mothers [48]. Another possibility is that our finding simply reflects the higher rates of mental disorder in mothers relative to fathers (38 vs. 18%), which may have decreased power to detect a significant relationship between paternal psychopathology and parent-dependent life events.

As predicted, maternal and paternal histories of mental disorder were significantly related to increased parent-dependent chronic adversities. The finding for mothers was consistent with the presence of a significant linear relationship between maternal self-reported distress and parent-dependent chronic adversities. Our study featured parents with a history of a range of different disorders including anxiety, depression and substance abuse. The present study could benefit from the inclusion of a structured diagnostic interview that includes current and lifetime diagnoses for adults (e.g., ADIS-IV-L [49]) to provide a more formal assessment of parent history of mental disorder. This would allow for investigation of the timing of clinical episodes experienced by both parents and children, and provide a test of parent-dependent stressors as potential mediators of the relationship between different forms of parental psychopathology and child anxiety. The cross-sectional design of the present study precludes any conclusions about the direction of the relationship between parent psychopathology, stressors and child anxiety. For example, parent psychopathology may increase the likelihood of parent-dependent chronic adversities such as interpersonal conflict or vice versa. Parents may view themselves as responsible for behaviour-dependent chronic adversities, increasing the distress caused by these stressors, which in turn may increase symptoms of psychopathology. Similarly, anxious children may elicit behaviour from parents that increases the likelihood of both stressors and parent distress. Thus, consistent with transactional models of symptoms and stress [17, 18], the relationship between child anxiety, parent behaviours and distress may be bidirectional, as children’s anxious vulnerability both shapes, and is shaped by parental expectations and behaviours. There may also be factors that contribute to both parent psychopathology and increased rates of parent-dependent chronic adversities, such as stressful work conditions. The mechanisms explaining how children’s anxious vulnerability, parent-dependent stressors and parent psychopathology interact to influence the development of child anxiety are likely to be varied and complex, and include the identification of direct, bidirectional and transactional relationships over time [50]. Longitudinal research is needed to clarify the nature and direction of relations between parental psychopathology, parent-dependent stressors and the timing of clinical episodes of anxiety in children.

The mechanism linking parental history of mental disorder to increased rates of parent-dependent chronic adversities is also unknown. Anxious parenting practices (e.g., overprotective parenting, low warmth, modelling of avoidant coping) are heightened in the presence of parental anxiety disorders, particularly when the family faces stressful circumstances [2, 51]. Future research could investigate factors that may underlie parental psychopathology and influence the likelihood of exposure to stressors related to parent behaviour, such as parent characteristics (e.g., neuroticism, insecure attachment style, anxiogenic cognitions, avoidant problem-solving), contextual factors which may result from parent behaviour (e.g., mate selection, marital relationship quality) and other situational characteristics, such as employment conditions, health, income and education [52].

The generalisability of our findings is limited by our small sample size, and the predominantly White Australian, middle class sample. Current findings need replication in a larger, more representative sample featuring ethnically diverse, low-income families. This study defined onset as the presence of anxiety symptoms plus associated impairment, consistent with the DSM-IV [29]. However, symptoms may be present for long periods before impairment becomes apparent and in fact, anxiety disorders are commonly preceded by similar temperamental expression [53]. Thus future research on the appropriate choice of diagnostic threshold is warranted, a complex issue reflected in the current debate about dimensional versus categorical approaches to the diagnosis of anxiety disorders in the DSM [54]. Another limitation is that mothers may have inaccurately recalled stressors due to memory, mood or personality biases. However, past research has shown a drop-off of recall of only 1% when employing similar features to PACE to aid recall [55]. Furthermore, retrospective report has shown good validity when clear definitions are provided in terms of what constitutes a life event/chronic adversity [56], as is the case with PACE. We attempted to minimise the possibility that maternal perception bias would result in the over-reporting of stressors through the use of independent panel judgements of whether reported experiences met criteria for a life event or chronic adversity. However, panel ratings were based on information provided by mothers and thus may still have been clouded by maternal perception bias. Furthermore, shared rater bias may have influenced findings for stressors, paternal and maternal psychopathology, as mothers were the sole informant for these variables. We considered maternal report appropriate given the young age of the children and mothers’ ability to provide information about parent health, work-related events and family finances. However, future studies could include father’s report of their current and past psychopathology, child anxiety symptoms and stressors or use official records (e.g., school and health records) to verify the dating and occurrence of events.

To the best of our knowledge, this study is the first to examine links between parental psychopathology, parent-dependent stressors and anxiety in children. This study has many strengths, including a matched pairs design to control for child age and gender, and the inclusion of interview assessment of parental psychopathology. Goodyer et al. [14] relied on parent self-report, so it is unclear whether their findings reflect mental disorder or more enduring aspects of parents’ personality. The use of a investigator-based life events interview allowed for the comparison of life events on the basis of maternal (subjective) and panel (contextual) impact ratings, the differentiation of life events from chronic adversities, and assessment of the independence of stressors from parent behaviour [57]. The inclusion of paternal history of mental disorder is important given theory emphasizing the importance of fathers, as well as the potentially differing roles of mothers and fathers in the etiology of anxiety [25, 26]. Fathers tend to encourage children to be brave and assertive, while mothers provide calm and comfort when children are distressed [26]. The mechanism linking child anxiety, parent-dependent stressors and parental psychopathology may, therefore, differ for mothers and fathers. For example, the presence of psychopathology in mothers may decrease their ability to soothe their child following stressor exposure and/or increase the likelihood of punitive, avoidant or distress reactions, while psychopathology in fathers may compromise their ability to model and encourage brave and autonomous behaviour when faced with challenging situations [54, 58].

The present study provides support for increased rates of parent-dependent chronic adversities prior to the recent onset of anxiety in children, and suggests that parental history of mental disorder dramatically increases children’s risk of exposure to parent-dependent chronic adversities. In contrast, parent-dependent negative life events were not significantly associated with an increased risk for the onset of anxiety. However, we cannot rule out the possibility that parent-dependent life events may not contribute to the onset of anxiety as our small sample size may have restricted the power of the present study to detect significant associations with life events. Anxious children who have a parent with a history of mental disorder may be at increased risk for the recurrence of clinical episodes due to a combination of genetic vulnerability to psychopathology and exposure to parent-dependent chronic stress. Our findings provide support for theories highlighting the influence of stressful experiences on anxiety in children [1, 2]. However, current findings suggest that these models should incorporate the distinction between these two types of stressors as well as mechanisms that may elucidate the pathways between parental psychopathology, parent behaviour-related stressors and child anxiety. Our findings indicate that interventions for anxiety in children should include support for parents, particularly those with a current or past history of mental disorder, to reduce parental behaviours that may contribute to a stressful family environment.
